# Looking beyond relative age to understand relative advantage and disadvantage in talent development

**DOI:** 10.3389/fspor.2024.1470944

**Published:** 2024-11-11

**Authors:** Liam Sweeney, Alfonso de la Rubia, Jamie Taylor, Christian Thue Bjørndal

**Affiliations:** ^1^ Department of Sport Science and Nutrition, Faculty of Science and Engineering, Maynooth University, Kildare, Ireland; ^2^ Deporte y Entrenamiento Research Group, Departamento de Deportes, Facultad de Ciencias de la Actividad Física y del Deporte, Universidad Politécnica de Madrid, Madrid, Spain; ^3^ School of Health and Human Performance, Faculty of Science and Engineering, Dublin City University, Dublin, Ireland; ^4^ Insight SFI Centre for Data Analytics, Dublin City University, Dublin, Ireland; ^5^ Grey Matters Performance Ltd., Stratford Upon Avon, United Kingdom; ^6^ Department of Sport and Social Sciences, Norwegian School of Sports Sciences, Oslo, Norway; ^7^ Child and Youth Sport Research Center, Norwegian School of Sports Sciences, Oslo, Norway

**Keywords:** athlete development, relative age effects, youth sport, talent identification, talent systems

## Abstract

In this perspective article, we argue for a broader consideration of relative advantages and disadvantages in youth sport; a lens that considers the complex biopsychosocial factors that influence athlete development beyond relative age. We begin with a brief overview of Relative Age Effects (RAEs), with a particular focus on the proposed underlying mechanisms, followed by a discussion of the cultural and organisational considerations and implications that talent systems must consider when implementing interventions to counteract RAEs. We conclude by proposing key directions for future research in respect to RAEs and talent development more broadly. We argue that there is a need to consider the highly complex nature of RAEs, but also that there are no clear solutions to the issue of RAEs in youth sports, and that proposed solutions may come with unintended consequences. This should encourage us to experiment more, not less**,** with diverse ways of providing meaningful sports experiences that promote learning, psychosocial development, and performance. We suggest an urgent need for greater practical and research focus on supporting coaches, as they have the greatest capacity to understand the needs of individual athletes. In addition, systemically working towards equal access to skilful coaches. We encourage a shift in focus beyond descriptive methodologies of RAEs toward transformative research methodologies that include action-based research, complex interventions that incorporate context-sensitive qualitative methods, and other participatory research approaches.

## Introduction

Talent development (TD) in sport is well established as a complex, dynamic, and typically non-linear process, with a range of biopsychosocial variables having the potential to impact a young athlete's progression (1, 2). In youth sport, national federations and their respective clubs are typically responsible for identifying and nurturing young athletes through this developmental process to the senior elite level (3). In some contexts, young athletes can be recruited into formalised TD systems from as young as five years of age (4). Early selection is proposed to maximise the long-term provision of significant developmental resources to the most talented athletes to facilitate their transition to the elite senior level (3, 5). However, evidence across several TD contexts indicates that the traditional identification and selection of young athletes may often lead to the over selection of those with early advantages (i.e., experiential, physical) relative to peers (6–9). This is of further significance given that junior and senior success in sport are not synonymous (10).

One extensively studied concept proposed as providing significant early advantage is advanced relative age (11–13). Relative age represents one's chronological age relative to the individual birthdate and competition cut-off date (9). In a sporting context, Relative Age Effects (RAEs) are a selection bias in favour of those athletes born earlier in the selection year or biannual competition cycle, who are chronologically older, at the expense of those born later in the selection year, in a given cohort. The proposed early advantage is a population level effect with significant evidence of selective TD system populations, prior to the elite level, slanted towards those youth athletes born in the first two quartiles of the year at the expense of those born in the third and fourth quartiles (3, 7–9, 14–17). For example, in an investigation of 1,212 male players aged 8–18 years from 17 professional soccer academies in the United Kingdom, players born in the first quartile were 49% of those selected, with players born in quartile four comprising just 9% (7). In the United Kingdom, U13 male 100 m sprint athletes born in the first quartile made up 48% of those selected, with those born in quartile four representing just 8% (18). In an investigation of the female American soccer talent system, youth players born in the fourth quartile of the year accounted for just 14% of all players selected at the club, national and international level (13). In a 16-year longitudinal study of the TD system in Spanish handball (organised in biannual cycles), relatively older U19 and U21 male and female players (Q1) accounted for 18% of those selected to compete internationally, compared to 8% of relatively young players (Q8) (19).

Despite contextual differences and variations between biological sexes, the disproportionate over selection of athletes born in the first two quartiles of the selection year appears relatively consistent (although with variation in magnitude) across a multitude of contexts in both male youth [i.e., (8, 9, 20)], and to a lesser extent, and female youth [i.e., (13)], spanning from local to international levels (3, 14, 16, 17, 19, 21). In some instances (e.g., male soccer in the United Kingdom), RAEs have been observed in early-mid childhood (7, 9). Indeed, RAEs may also be present at earlier stages of the pathway, before selection into formalised TD systems occur (22, 23). Further, RAEs have been shown by some authors to be present at the youth level but to dissipate at the senior level (24). Other authors, however, have shown that RAEs may too persist at the senior level (25). Moreover, some research underlines the relevance of RAEs in the selection and re-selection of players for U17, U19 and even senior national teams (25, 26). These findings have sparked a multitude of investigations to explore the mechanisms underpinning RAEs, with a view to provide recommendations to promote developmental equity within TD systems (27).

### Mechanisms underpinning relative age effects

Several factors have been proposed to cause RAEs, the majority of which relate to factors associated with differences in age and experience. Musch and Grondin (11) suggested that the mechanisms underpinning RAEs are multifactorial and related to a combination of physical, cognitive, emotional, motivational, and social factors. In discussions relating to male youth soccer, it has been suggested that RAEs may be attributed to a variety of factors including: age associated differences in sport knowledge and understanding, decision making, cognition, and psychosocial development (9, 28). Recently, Fitzgerald et al. (20) discussed how RAEs may be partially related to age-associated differences in neural development. With the development of the brain and nervous system showing rapid changes during infancy and early childhood, and a single chronological year representing a difference of more than 20% total neural development from age 2–3 years, this can present an advantage for a relatively older athlete within a given cohort (20). On the other hand, Wattie et al. (29) considered that the existence or non-existence of a RAE must necessarily be understood from how individual, task, and environmental constraints facilitate performance in the specific sport context. Rather than being attributable to several direct factors, McCarthy et al. (30) suggest that RAEs are a more complex population-level consequence of a constellation of factors that are difficult to measure or quantify. In short, RAEs being something that we cannot directly attribute to a defined set of tangible factors, particularly as the extent of proposed advantages (and the factors providing the advantage) differ between individuals and contexts (30). Moreover, at the individual level, some relatively older athletes may not benefit from advantages at all (28, 30–32). As one example, in Swiss female youth TD programmes, relatively older alpine skiers and tennis players are overrepresented and appear advantaged, yet relatively older snowboarders, fencers and table tennis players are underrepresented and appear disadvantaged (33). Data in female Italian soccer show the same proportion of relatively older and younger players represent the international team at the senior level in absolute terms (34). Further, Kelly et al. (35) show that relative advantages or disadvantages in sport go beyond just age and experience. In this regard, contextual elements of the athlete's environment and an interaction with their sporting experiences influence relative advantages in sport, and subsequent progression through the pathway (35). Considering the influence of context, De La Rubia et al. (36) demonstrated that advantages/disadvantages associated with RAE influenced competition performance. This, ultimately, highlights the contextually driven complexity of RAEs with so much still unclear, despite 40 years of research in sport. At this point, it is important to acknowledge that the vast majority of investigations focussed on RAEs in sport have been dominated by research specific to males (37). Indeed, Curran et al. (37) note that of the research focussed on RAEs or biological maturation published between 1999 and 2019, 63% is specific to male populations. Indeed, this is a broader issue in the TD research, whereby female specific investigations represent just 9% of the data (37). We urge the reader to acknowledge the sex gap in the RAE literature, and TD literature more broadly, considering such implications when reading our article.

### Independence of relative age and biological maturity

Although it was initially suggested that RAEs may be attributable to differences between individuals in biological maturation (and the associated physical, functional and physiological advantages conferred by advanced biological maturity) (38, 39), there is now a substantial evidence base to highlight that biological maturation and relative age are independent and uncorrelated (8, 9, 28, 31). Indeed, there is data showing that the relatively oldest selected athletes within a given cohort have been amongst the more delayed in biological maturity (8, 9, 17). Biological maturation is the process of progression toward the mature adult state and is defined in terms of status, timing, and tempo (40–42). Significantly, youth of the same chronological age can differ up to 6 years in skeletal age and somatic maturity, both of which are established indices of biological maturity status and timing (31, 43–45). The individual differences in biological maturation between youth of the same chronological age are primarily attributable to inherent genetic factors, although the influence of environmental factors (e.g., chronic malnutrition, disease, climate) have also been noted, to a lesser extent (46). Note that none of these factors relate to differences in age and experience between individuals within the same chronological age cohort.

Advanced biological maturity provides several distinct physical, functional and physiological sporting advantages (e.g., increased stature, body mass, lean muscle mass, peak force production, absolute muscular strength and power) (40, 47–52) (although varying markedly in magnitude between biological sexes, with a substantially disproportionate evidence base in favour of male youth), none of which are directly correlated to advanced relative age (8, 9, 17, 20, 31). The selection advantages conferred by advanced biological maturity in youth team sport are typically observed at the onset of puberty (8, 9), whereas those associated with relative age have been observed in childhood (7, 9). For a more substantial discussion on the distinct differences between biological maturity and relative age, see the work of Towlson et al. (31).

### Cultural and organisational considerations

The landscape of TD and elite sport systems varies significantly across different cultural and organisational contexts, requiring tailored strategies that recognise these differences without uncritically adopting imported solutions (53). For instance, in the United Kingdom, the centralised approach and focus on early engagement within soccer academies contrasts sharply with the more dispersed, multicentric and grassroots-based approaches observed in the Scandinavian countries (21). Whilst early selection and a focus on early engagement affords the opportunity to shape the developmental journey, a consequence is that large numbers of young athletes are excluded access to such systems from early ages (27). On the other hand, a multicentric and grassroots-based approach promotes a breadth of engagement opportunities but presents additional challenges to the shaping of athlete experience, particularly in ensuring quality coaching and specialised support (27). Strategies to manage unequal opportunities for access and learning due to relative age should, therefore, be tailored to specific contexts, whilst remaining mindful that regardless of specific approach, there may be unintended consequences as a result of implementation. In selection-based TD systems, one such strategy is to allow for flexible entry points and progression routes, ensuring that grassroots settings are of sufficient quality so that so called; “late developers”, those subject to relative early disadvantage, are not entirely excluded (54).

Mitigating inequalities related to relative age will also differ between sports depending on the age of selection to the system. For example, as a contrast to the pre-adolescent first selection point in professional English soccer, in rugby union, large cohorts of players are first selected to formal academies from 15 years of age, with evidence of significant subsequent selection and deselection.^1^ Similarly, TD systems in Spanish handball carry out the first selection of player cohorts around the age of 12–13 years at the regional/national level (the time at which athletes leave school sport) and at the age of 15–16 years to compete in international contexts. Again, this contrasts to European soccer, whereby players may compete internationally from 14 years (17, 32, 55). Cultural differences within the same sport, both across countries and local contexts, may also dampen or amplify issues related to relative age, and even biological maturation, such as whether the distinct culturally significant ways of playing the sport favours physical or refined technical-tactical prowess (56, 57). A final consideration that organisations need to address is the disruption of social relationships with peers in sport. Given that the quality of social relationships among peers are essential to continued participation, learning and performance in organised sports (58), any strategy used to target advanced relative age should only be implemented as complementary measures and carefully adopted in conversation with the individual children themselves. Creating opportunities for periodic practice and competition with relatively older athletes (i.e., playing up), for example, may aid development, provided that the children themselves perceive it as appropriate, meaningful for development, challenging, encouraging, and fun (59–62). Additionally, alternating age categories used in national and international competition so that players can adapt to playing with both relatively younger and older players, and experience different relative levels of individual performance, should also be considered (36). By acknowledging and addressing these cultural and organisational differences, and by implementing strategies to mitigate inequalities related to relative age, sport organisations can create more equitable and effective TD systems that not only enhance performance, but also support the psychosocial development of young athletes.

### Implications for relative advantage and disadvantage in talent systems

The identified distinction between relative age and biological maturation points to differential means of intervention or non-intervention. This, in turn, presents significant considerations with the implementation of strategies aimed toward optimising talent system processes or promoting equity of selection. In the case of relative age, significant research attention has been focused toward generating selection practices that lead to greater numbers of later born athletes being selected (12, 63). Yet it appears that across some sporting settings, those selected few born later in the selection year, *if retained within the system*, may also benefit from exposure to the demands associated with competing with relatively older athletes (64, 65). However, it should be noted that in absolute terms, relatively younger athletes were still underrepresented at the senior level in these studies (66, 67). There is also somewhat similar evidence presented in the context of biological maturation (68). Further, there is a need for due consideration to the potential for non-selection or dropout of relatively younger athletes at the youth level, but also the notable dropout of relatively older athletes at latter stages of the pathway (69). As such, not only do we need to consider the highly complex nature of RAEs, but also the lack of systematic evaluation of the impact of interventions that have sought to alter its magnitude. More broadly, consideration must be given to the potential unintended consequences if a given intervention is “effective”.

There is then a need to make decisions related to the bar at which interventions might be necessary. As there is growing pressure on sporting organisations to present equitable access to talent systems, there becomes a question for National Sporting Organisations (NSOs) to consider the extent to which individual National Governing Bodies (NGBs) are responsible for wider societal issues such as equitable access to facilities and equipment, especially in sports with high barriers to entry based on cost. As an example, whilst English Premier League soccer academies are often criticised based on early selection practices, it appears that selected players represent a highly socially diverse population (70). Yet, the solution of providing extensive support to an economically and socially diverse population, including transport, from an early age is clearly beyond the reach of nearly all other contexts. In similarly resource rich systemic contexts, we should be looking to capture longitudinal data that might provide proxies to understand performance related challenge factors (e.g., biological maturation), population trends (e.g., RAEs), and non-performance relevant factors (e.g., proxies for socio-economic status, such as post/zip code). These types of data should inform systemic choices. Alternatively, in a system whereby selection is delayed until such a point whereby the dynamics of adolescence are dampened, without access to high quality coaching and support, we may simply be creating a situation whereby those with access to advantages such as good coaching and early perceptions of competence are simply further advantaged, at least at the first point of selection. That is to say that there are no contextless solutions that can be deployed against the complexity of problems presented by relative advantages and disadvantages (53, 71).

The alternative proposal is that as increasing inequity is identified across youth sport, perhaps the most equitable thing that any individual can have is access to high quality coaching. Whilst the temptation for sporting organisations might be to seek out increasingly elaborate forms of control through regulation, our proposal is to put sustained, genuine effort into the development and leadership of coaches. A change in focus that would require a profound shift in the focus of resourcing for many sporting organisations. To be clear, this is not the somewhat stereotypical research implication that “coaches should be educated to understand RAEs”. Despite 40 years of research, we do not appear to be in a position to offer implications for the individual coach specifically related to RAEs. This proposal is far more radical; one that aims calls for the lofty aim of equal access to high quality coaches that can individualise based on the complexity of relative advantage and disadvantage, with a long-term view of development. As such, our proposal for this special edition is that the scope and spectrum of research in sport needs to reach beyond RAEs toward understanding a breadth of relative advantage and disadvantage if it is to genuinely enhance practice.

## Directions for future research

In respect to RAEs to date, arguably, we are yet to move the research base forward beyond acknowledging its existence through analysing the proportion of TD system populations slanted towards a particular quartile and how that may vary in magnitude across different sports and contexts, or in the transition from the youth to senior level. One primary reason for this is likely due to the fact that the overwhelming majority of research into RAEs has been observational and descriptive in nature. Indeed, there is still no clear understanding of the mechanisms underpinning RAEs. Yet, with a push for researchers to implement interventions to counteract RAE's (12, 63, 72, 73), how can developmental solutions be implemented if there are no clear mechanisms to target? Based on the conceptualisation we reference in this paper, relative advantages or disadvantages in the youth sport domain likely qualify as being a “wicked problem”, a problem that changes based on intervention (74) and is therefore immune to simplistic solutions.

Despite a plethora of research investigating developmental interventions that attempt to manipulate RAEs, promote developmental equity, increase opportunity or adjust the interpretation of performance based upon relative age [e.g., (12, 63, 72, 73, 75)], there are still no prospective and longitudinal investigations that support the implementation of any intervention in respect to long-term TD in sport. This is not to say that interventions do not have the capacity to enhance developmental equity in the long term, but that there is currently no evidence in their favour over the long term. Such findings are not possible without utilising a range of methods prospectively; research that is simply yet to be conducted. This research should involve both large sample sizes across contexts and also socially situated phenomenological work to understand the breadth of developmental experience between individuals. One promising area for research is also the implementation of flexible methods for organising practice and competition within sports clubs and organisations, moving beyond the traditional age-segregated groups. This may include, for example, flexible and consistently evolving age groups, as well as moving athletes between and across age groups based upon specific and individually identified athlete needs (61, 76). This allows for the exposure of athletes to those that are both older and younger and an overall diversity of experience. In essence, diversifying learning experiences in sport may offer greater potential compared to the homogenized training methods and experiences common in many academy or talent pathway systems to date.

By promoting equitable practices, we can support the development of young athletes by contributing to the implementation of more sustainable athlete development models. However, this pathway must be compatible with the possibility of offering those with ambitions for sports careers focused on high performance just that in the same context (e.g., club). Thus, the stage at which the domain becomes more TD towards high performance focused, when inclusion becomes less of *a priori*ty, should be an active strategic decision and one that informs research. As demonstrated, there are no clear solutions to the issue of relative age in sports, and all proposed solutions come with unintended consequences. This reality should encourage us to *experiment more, not less,* with diverse ways of providing meaningful sports experiences that promote learning, psychosocial development, and performance. To achieve this, transformative research methodologies are encouraged, including action research, complex interventions that incorporate context-sensitive qualitative methods, and other participatory research approaches.

## Footnotes

^1^Shelly A, Behan S, Taylor J, MacNamara Á, Sajwan V, Scriney M, et al. The impact of high-potential status on progression and conversion within a male rugby union talent system. *J Sports Sci.* (In Review).
